# *Salmonella* Serovars Distribution in Poultry Meat and Genetic Characterization of Antimicrobial-Resistant Isolates: A Five-Year Monitoring in Sicily

**DOI:** 10.3390/ijms27219876

**Published:** 2026-11-04

**Authors:** Annamaria Castello, Vincenzina Alio, Ruggero Baviera, Giuseppa Oliveri, Valeria Gargano, Maria Rita Schiavo, Antonina Zimmardi, Cinzia Cardamone, Rosa Alduina, Antonella Costa

**Affiliations:** 1 Experimental Zooprophylactic Institute of Sicily A. Mirri, 90129 Palermo, Italy; vincenzina.alio@izsssicilia.it (V.A.); giuseppa.oliveri@izssicilia.it (G.O.); valeria.gargano@izssicilia.it (V.G.); mariarita.schiavo@izssicilia.it (M.R.S.); antonina.zimmardi@izssicilia.it (A.Z.); cinzia.cardamone@izssicilia.it (C.C.); antonella.costa@izssicilia.it (A.C.); 2 Department of Biological, Chemical and Pharmaceutical Sciences and Technologies Department, University of Palermo, 90128 Palermo, Italy; ruggero.baviera@community.unipa.it (R.B.); valeria.alduina@unipa.it (R.A.)

**Keywords:** *Salmonella*, AMR, ARGs, third-generation cephalosporins, poultry

## Abstract

Salmonellosis is one of the most frequently reported zoonoses, and broiler meat/meat products are among its main vehicles of transmission. Continuous monitoring on *Salmonella* serovars and the spread of antimicrobial resistance is essential for epidemiological surveillance. From 2021 to 2025, a total of 350 poultry samples were collected in Sicily and analyzed for *Salmonella* spp. detection, serotyping and antimicrobial resistance (AMR) phenotyping. Overall, 101/350 samples tested positive for *Salmonella* spp. Eight serovars were identified with *S.* Infantis being the most prevalent, followed by *S.* Kentucky, *S.* Enteritidis, *S.* Strathcona, *S.* Agona, *S.* Corvallis, *S.* Toulon, *S.* Virchow. The following resistance percentages were revealed: 78.7% (144/183)—tetracycline, 68.9% (126/183)—trimethoprim-sulfamethoxazole, 51.9% (95/183)—penicillins, 35.0% (64/183)—third-generation cephalosporins, 33.3% (61/183)—aminoglycosides, 4.9% (9/183)—chloramphenicol, 2.2% (4/183)—fluoroquinolones, 0% (0/183)—imipenem. The following genetic determinants were searched in a selected subset of 46 isolates resistant/intermediate resistant (R/I) to third-generation cephalosporins or fluoroquinolones: *bla*_CTX-M-1_, *bla*_CTX-M-2_, *bla*_CTX-M-4_, *bla*_CMY-2_, *bla*_TEM_, *bla*_OXA_, *bla*_SHV_ (third-generation cephalosporins); *qnrA*, *qnrB*, *qnrC*, *qnrD, qnrS*, *qepA*, *oqxAB*, *aac(6′)-Ib* (fluoroquinolones) and *intI1*. High proportions of R/I isolates (78.6% for third-generation cephalosporins, 100% for fluoroquinolones) carried at least one of the resistance determinants. All of them carried *intI1*. Our data confirm that poultry meat and poultry-based foods represent important vehicles of *Salmonella* and that *S*. Infantis is the predominant serovar. The high rate of AMR is of concern for human safety and environmental health, especially referring to the highest priority and critically important antimicrobials (hpCIAs) such as third-generation cephalosporins.

## 1. Introduction

*Salmonella* spp. is a rod-shaped, Gram-negative, facultative anaerobic, non-spore-and biofilm-forming bacterium belonging to *Enterobacterales* [1]. The genus *Salmonella* includes two species: *Salmonella bongori* and *Salmonella enterica*; the latter is further divided into six subspecies that include over 2600 serovars, differentiated by host adaptation, specific disease syndromes and geographic and/or source distribution [2]. Specifically, non-typhoidal serovars (NTS) are ubiquitous enteric pathogens of wild and domestic animals that play an important role as reservoir of this pathogen [1,3]. Both indirect and direct transmission to humans is possible, even though the majority of illnesses are foodborne [1]. This is due to the capacity of *Salmonella* to grow and survive at a wide range of temperatures (between 5 °C and 47 °C [4] and at −20 °C and 4 °C for up to 1 year in food matrices [5]). According to the last report on zoonoses released by the European Food Safety Authority (EFSA) and the European Centre for Disease prevention and Control (ECDC), in 2024 salmonellosis was the second-most reported zoonosis in humans, *Salmonella* spp. was the most common cause of multi-country foodborne outbreaks, with broiler meat/meat products among the most frequently implicated vehicles and *Salmonella* Infantis and *Salmonella* Enteritidis listed as the first and the second most reported serovar, respectively, in broilers and broiler meat [6].

Symptoms of non-typhoidal salmonellosis usually last between 2 and 7 days but bacteria can be shed via feces for over one month. The disease is usually self-limiting in immunocompetent hosts, while severe symptoms can be observed and can escalate to life-threatening conditions among high-risk groups, such as babies and children under five years, the immunocompromised and the elderly [1,4,7]. When severe symptoms are observed, the use of antimicrobials such as fluoroquinolones and third-generation cephalosporins is recommended [7]. In *Enterobacterales*, such as *Salmonella*, resistance to fluoroquinolones is mainly related either to mutations in the chromosomal genes of quinolone resistance-determining regions (QRDR), such as the substitutions D87G or S83Y in *gyr*A sequence [8,9], or to plasmid-mediated quinolone resistance (PMQR) genes, such as *qnr*A-D, *qnr*S [10], *qep*A, *aac(6′)-Ib-cr*, and *oqx*A-B [8,11]. The main mechanism of resistance to third-generation cephalosporins is the acquisition of genes (*bla* genes) that belong to the TEM, SHV, CTX-M families and include multiple variants. These gene families include variants encoding extended-spectrum beta-lactamases (ESBLs), which hydrolyse first-, second-, third-generation penicillins and cephalosporins [12], and are frequently located on plasmids [13]. ESBL genes have been identified in bacteria isolated from food-producing animals and various food products, including poultry and poultry meat [14,15], highlighting how this production chain can play a key role as a reservoir for these ARGs and their spread in the environment.

The spread of antimicrobial resistance (AMR) poses a serious threat to food safety and human health, particularly when infections caused by resistant bacterial clones become more difficult to treat [16]. According to the EU’s joint inter-agency antimicrobial consumption and resistance analysis (JIACRA) reports, antimicrobial use in animal husbandry and possibly also in humans seems to facilitate the dissemination of particularly resistant bacterial clones, among which highly ciprofloxacin-resistant and MDR (multidrug resistant, strains featuring resistance to antibiotics belonging to at least three different classes) *S.* Kentucky and ESBL-producing *S.* Infantis are of particular concern, both poultry-related and among the top serovars in human *Salmonella* infections in the EU/EEA [17]. Antimicrobial resistance genes (ARGs) can be transferred among bacteria belonging to the same or to different species and the cell-to-cell contact in bacterial biofilms can contribute to horizontal gene transfer (HGT) enhancement [18,19]. In the context of HGT, various types of mobile genetic elements (MGEs) may function as carriers of AMR determinants and enhance the rate of AMR dissemination. In particular, class I integrons perform site-specific genetic recombination, playing a key role in the genetic rearrangements for the acquisition of ARGs, their expression and their subsequent intra- and inter-species dissemination [20]. Integron integrases can be coded by three genes, namely *intI1*, *intI2*, and *intI3*, with *intI1* being the most frequently reported in association with various ARGs, in particular among *Enterobacterales* [21,22]. This study aimed to investigate the prevalence of *Salmonella* spp. isolated from poultry meat and poultry-based foods sampled in Sicily between 2021 and 2025 to delineate the antimicrobial resistance phenotype of the isolates, and to characterize their AMR genetic profiles, in particular referring to the genetic determinants for resistance to fluoroquinolones and third-generation cephalosporins, whose effectiveness is crucial against particularly severe symptoms or notably resistant clones. Considering the marked adaptability of non-typhoidal *Salmonella* to the environment, the spread of this microorganism through feces and via a wide range of vectors/carriers, the strong ability of certain serovars (such as *S.* Infantis) to acquire and transfer ARGs, the data collected in this study are essential for an effective monitoring of *Salmonella* AMR and the evaluation of its implications for food safety, public and environmental health. Similar studies carried out previously and/or in different geographical areas of Italy lack results related to the molecular analyses conducted in the present study. Assessing how many clones that are resistant to fluoroquinolones and cephalosporins carry the genetic determinants searched in this study can provide an additional perspective on how the afore mentioned phenotypes might spread among *Salmonella* spp. as well as to other potentially pathogenic bacteria, encountered both at the food level and at any stage along the production chain.

## 2. Results

**Figure 1:**
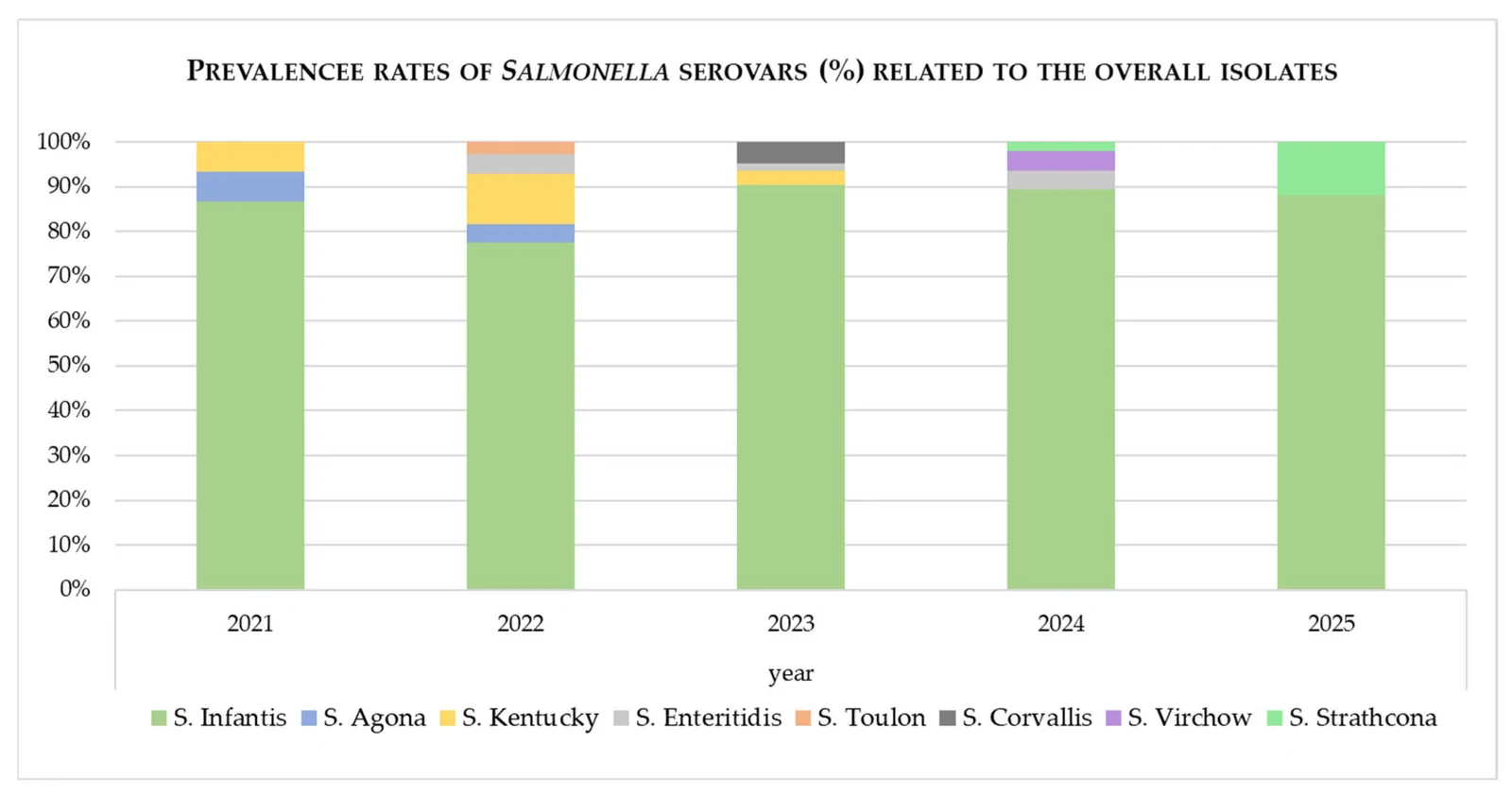
Prevalence of *Salmonella* spp. serovars (%) related to the overall isolates detected each year between 2021 and 2025.

Out of the 350 samples analyzed, 101 (28.9—confidence interval 95% = 24.1–33.6) tested positive for *Salmonella* spp., among which were 14/59 (23.7%) in 2021, 26/71 (36.6%) in 2022, 21/73 (28.8%) in 2023, 22/71 (31.0%) in 2024 and 18/76 (23.7%) in 2025. A significant difference among annual prevalences (*p* value < 0.05) was observed between 2021 and 2022 and between 2022 and 2025. The remaining between-year comparisons were not statistically significant. Referring specifically to each sample type, the highest contamination percentage was recorded among frozen meat preparations (6/7–85.7%; *p*-value < 0.05), followed by skins (38/87–43.7%; *p*-value < 0.05), meat pieces/carcasses (52/234–22.2%; *p*-value < 0.05) and raw meat preparations (4/21–19.0%). Referring to 61/101 positive samples, more than one sample unit tested positive for *Salmonella* spp., and 6/61 samples were simultaneously contaminated by multiple serovars (Table S1). A total number of 253 *Salmonella* spp. isolates (30 in 2021, 71 in 2022, 63 in 2023, 47 in 2024, 42 in 2025) out of the 101 positive samples were collected and serotyped. *S.* Infantis was the predominant serovar, accounting for 85.8% (217/253) of the isolated strains, followed by *S.* Kentucky (12/253–4.7%), *S.* Enteritidis and *S.* Strathcona (6/253 each–2.4%), *S.* Agona (5/253–2%), *S.* Corvallis (3/253, 1.2%), *S.* Toulon and *S.* Virchow (2/253 each–0.8%). Referring to each year individually, a greater variability of circulating serovars was observed in 2022 (5/8 serovars) compared to 2021 (3/8 serovars), 2023 and 2024 (4/8 serovars) and 2025 (2/8 serovars). The serovars detected showed a different temporal distribution, in particular: *S.* Infantis was detected along the whole period taken into consideration, with *S.* Strathcona apparently detected for the first time in poultry meat during the study period in 2024 and becoming the only serovar circulating in these matrices together with *S.* Infantis but at a very low percentage (5/42 isolates, detected in 1/18 samples tested). *S.* Kentucky was detected from 2021 to 2023, *S.* Agona only in 2021 and 2022, *S.* Enteritidis from 2022 to 2024, *S.* Corvallis and *S.* Virchow were detected only once, in 2023 and 2024, respectively (Figure 1).

Based on the results obtained from serotyping, a subsample of 183 isolates were subjected to AMR phenotyping. All of them were R/I to at least one class of antimicrobials and 77.0% (144/183) were resistant to at least one agent in three or more different antibiotic classes (MDR). The resistance percentages obtained are reported in Table 1.

The resistance percentages obtained from each of the top five serovars individually are reported in Table 2. Given the small sample size of some isolates’ groups, confirmation over a greater number of samples is required. This is difficult to carry out because *S.* Infantis is generally the main and most widespread serovar.

Genetic determinants for AMR were searched in a selected subset of 46 of the 253 isolates, which were R/I to third-generation cephalosporins and/or fluoroquinolones. Among the 46 isolates selected, 38/46 were R/I solely to third-generation cephalosporins, 4/46 were R/I to third-generation cephalosporins and to fluoroquinolones, 4/46 were R/I solely to fluoroquinolones. The molecular results reported below refer exclusively to this selected subset.

Forty-two *Salmonella* isolates R/I to third-generation cephalosporins were screened for ESBL- and AmpC-encoding genes. Overall, 33/42 isolates (78.6%) tested positive for at least one of the screened β-lactamase genes. Specifically, *bla*_CTX-M-1_ and *bla*_CTX-M-2_ genes were detected in 31 and 29 isolates, respectively, with co-occurrence of both *bla*_CTX-M-1_ and _2_ observed in 28 of the 33 ESBL-positive isolates. Notably, none of the screened isolates carried plasmid-mediated AmpC genes (*bla*_CMY-2_ or *bla*_DHA_) using the primer sets employed in this study. The remaining 10 isolates, which were negative for both ESBL and AmpC genes, were further screened for other β-lactamase genes. Among these, a single isolate was positive for *bla*_TEM_, whereas *bla*_OXA_ and *bla*_SHV_ were not detected in any of the tested strains.

Twelve isolates were R/I to at least one fluoroquinolone, with one being resistant to ciprofloxacin and intermediately resistant to levofloxacin. They, except for four isolates that were no longer viable, were screened for plasmid-mediated quinolone resistance (PMQR) determinants. No PMQR (*qnrA*, *qnrB*, *qnrC*, *qnrD*, *qnrS*, *qepA*, *oqxAB*) genes were identified in the eight isolates available for molecular analysis. No amplification products were obtained for *aac(6′)-Ib*; therefore, no subsequent characterization of the cr variant was performed. Sequence analysis of the quinolone resistance–determining region (QRDR) of the *gyrA* gene revealed that the Asp87Gly (D87G) substitution was the most prevalent mutation, occurring in 7/8 isolates (87.5%; Table S2). Among D87G-carrying isolates, six were intermediately resistant to ciprofloxacin while remaining susceptible to levofloxacin, and one isolate was susceptible to ciprofloxacin but resistant to levofloxacin. The Ser83Tyr (S83Y) substitution was identified in a single isolate, which exhibited a ciprofloxacin-resistant phenotype. All isolates subjected to molecular screening tested positive for the class 1 integron integrase gene (*intI1*). A detailed overview of antimicrobial susceptibility phenotypes and the corresponding genetic determinants is provided in Table 3.

## 3. Discussion

Here, we report the results of a five-year investigation about the prevalence, serovar circulation, and AMR characterization of *Salmonella* spp. in poultry meat/poultry-based food samples collected in Sicily. The high percentage of frozen samples found to be contaminated with *Salmonella* spp. (85.7%) highlights how even frozen foods, if handled improperly, can become reservoirs of the bacterium and a vehicle for cross-contamination during food handling. However, given the small size of this sample category (n. = 7), our results should be confirmed on a larger number of samples, for an appropriate understanding of the actual spread of viable Salmonella among these matrices. The overall prevalence of *Salmonella* spp. was 28.9%, similar to previous study periods covered in the same region [23]. The lowest prevalence rates were registered in 2021 and 2025 (23.7%), while the highest prevalence rate was registered in 2022 (36.6%; *p*-value < 0.05). Comparing our results with others, the prevalence recorded in 2022 is comparable with data from Apulia and Basilicata regions (36.1%) [4], while a great variability in terms of the overall prevalence reported is observed comparing our data with others, both from Italy as a whole and from nearby geographical locations within the same country. Indeed, the percentages of our study are higher than those reported in Italy between 2016 and 2020, with an overall prevalence of 7.49% [4] but lower compared to the overall prevalence of 35.6% recorded between 2021 and 2023 in Apulia and Basilicata regions [4] and to the overall prevalence of 86.7% recorded between 2011 and 2021 in Campania and Calabria regions [24]. The great variability in terms of distribution and prevalence is a well-known aspect of *Salmonella* spp., widely reported in the literature also when comparing data from different EU countries and at a global level [6,25,26]. Various factors may influence this variability, such as geographic location, livestock density and farming type, slaughtering and preparation culture, food production practices, as well as control measures at the farm level and vaccination programs applied in breeder flocks [4,7,26].

In line with data reported in the literature, *S.* Infantis was the predominant serovar (85.8% of the isolated strains), while others were detected with very low percentages, specifically *S.* Enteritidis and *S.* Strathcona (2.4% each), *S*. Agona (2%), *S.* Corvallis (1.2%), and *S.* Toulon and *S.* Virchow (0.8% each). Indeed, since 2010 *S.* Infantis has emerged and has traced an increasing trend in Asia [27,28], America [29,30], and Africa [31], becoming the primary serovar isolated in broiler production in Europe since 2014 and among the most frequent cause of human salmonellosis both in Europe [6] and at a global level [32]. The increasing prevalence of *S.* Infantis in poultry meat could be a result of the implementation of vaccinations against *S.* Enteritidis and *S.* Typhimurium in recent years [4]. Referring to *S.* Enteritidis, our results are in line with other reports from southern Italy [4].

Interestingly, between 2024 and 2025, two samples tested positive for *S*. Strathcona, a very rare serovar recently associated with multi-country outbreaks involving 17 European countries and vehiculated by small tomatoes from Sicily [33,34]. As far as we know, the current scientific literature does not report the presence of this serovar in poultry. Considering the relevance it has gained lately in terms of human health and food safety, our results certainly highlight the need to closely monitor the circulation of *S*. Strathcona, in order to promptly detect any potential spread of this serovar in matrices other than the plant-based ones. Considering that *S*. Agona is mostly prevalent in turkey meat chains [6], the presence of this serovar may be due to cross contaminations occurring in the working environments. About 5.9% of the positive samples were simultaneously contaminated by two different serovars. As suggested by Di Taranto et al. [4], the existence of multiple serovars in the same sample might be indicative of simultaneous infection of broilers through one or multiple sources (i.e., food, water, environment, etc.) or that meat can be contaminated multiple times by different serovars along the different steps of the food chain (i.e., slaughtering, handling, etc.).

The increasing frequency of *S.* Infantis illnesses is further complicated by the spread of resistant and multidrug-resistant (MDR) strains, including those capable of producing *β*-lactamases [32]. A key role for the spread of resistant and MDR clones is attributed also to the widespread, prolonged, and inappropriate use of antimicrobials in poultry and other animal farms for therapeutics, prophylaxis and growth promotion [4,35].

Regarding the AMR phenotyping of isolates, our study confirms high resistance of *Salmonella* spp. to nalidixic acid (82.0%), tetracycline (78.7%), trimethoprim-sulfamethoxazole (68.9%), penicillins (51.9%) and third-generation cephalosporins (35.0%), even reporting resistance percentage values slightly different to others [4,7]. Despite the ban imposed in 2006 by the European Union on the use of tetracycline as a growth promoter, high resistance to this molecule is still widely reported in the literature [14,18,23]. This could be due to its wide use, together with sulfamethoxazole and ampicillin, in veterinary medicine for treating infections in production animals [36,37].

Referring to fluoroquinolones and chloramphenicol, we recorded low resistance percentages (2.2% and 4.9% respectively). Opposite results were reported by Montone et al. [7], referring to both fluoroquinolones (56.2%) and chloramphenicol (45.7%), while Di Taranto et al. [4] obtained opposite results referring to fluoroquinolones (95.3%) and similar ones referring to chloramphenicol (3.9%). The use of chloramphenicol is banned in food-producing animals in all the member states of the European Union. The illegal use of these antimicrobials in farms randomly subjected to sampling was suggested [7] as a possible explanation for the high levels of resistance reported in some geographical areas compared to others [38]. However, this consideration is speculative and cannot be verified. Furthermore, multiple factors can influence the variable accumulation and bioavailability of this antibiotic in a specific ecological niche, such as soil composition, manure addition and bacterial species of the soil microbiota [39] with a direct effect on the selective pressure exerted on bacteria living in that niche.

Fluoroquinolones and third-generation cephalosporins are classified as highest priority critically important antimicrobials (hpCIAs) in human medicine and often constitute first-line treatment for invasive salmonellosis in humans. The emergence of resistance to these hpCIAs could lead to therapy failure, with severe public health consequences [4]. Further analyses were performed for the detection of genetic determinants related to the afore-mentioned phenotypes. A high percentage of the isolates R/I to third-generation cephalosporins subjected to molecular analyses (78.6%) carried at least one among *bla*_CTX-M-1_, *bla*_CTX-M-2_, and *bla*_TEM_. The *bla*_TEM_ variant was not further characterized; therefore, its detection alone does not establish the presence of an ESBL allele.

The genetic determinant detected most frequently was *bla*_CTX-M-1_, frequently associated with *bla*_CTX-M-2_ (90.3% of the bla_CTX-M-1_ positive isolates). All isolates resistant or intermediate (R/I) to fluoroquinolones and subjected to molecular analyses carried a single QRDR mutation, specifically the D87G substitution and with one isolate exhibiting an S83Y substitution in the *gyrA* gene. Both mutations have been previously reported in fluoroquinolone-resistant Salmonella [40]. These results confirm that fluoroquinolone-resistant *Salmonella* isolates are mostly associated with point mutations in QRDR [40]. According to the literature, the co-occurrence of QRDR and PMQR usually potentiate the fluoroquinolone-resistant phenotype [41,42]. None of the isolates tested positive for the PMQR determinants searched in this study. However, negative results for targets lacking positive controls should be interpreted cautiously. The detected QRDR mutations might explain how the isolates were either resistant or intermediately resistant to one of the two fluoroquinolones tested. Further analyses should be performed to confirm this hypothesis, including a greater number of isolates resistant to fluroquinolones.

Class 1 integrons play a key role in the genetic rearrangements for the acquisition of ARGs and their subsequent intra- and inter-species dissemination [20]. Although all the isolates subjected to molecular analyses carried the class 1 integron integrase gene (*intI1*), integron functionality and the presence of specific resistance gene cassettes were not assessed. Considering that class I integrons enable the acquisition of co-occurring ARGs, conferring resistance to aminoglycosides, sulphanilamides, quinolones and beta-lactams, except imipenem [21], and that the isolates testing positive only for *intI1* were R/I to aminoglycosides and/or trimethoprim/sulfamethoxazole (Table 3), further analyses should be performed to assess whether those isolates carry specific genetic determinants for resistance to aminoglycosides or sulphanilamides. Although *intI1* plays a key role in the dissemination of co-occurring ARGs from resistant to susceptible bacteria, it has also been reported to increase the rate of ARG acquisition when detected in “receiver” isolates [21]. For this reason, the widespread presence of the *intI1* gene among the isolates highlights the potential risk of increasing dissemination of AMR and MDR determinants, with worrying implications for the future management of infection treatment. Further studies would be desirable to demonstrate the actual occurrence of horizontal gene transfer among the isolates characterized, and to provide additional data for the effective assessment of the risk associated with such events.

The present study focused on resistance to critically important antibiotics, such as fluoroquinolones and cephalosporins; nonetheless, colistin resistance was also assessed on a subsample of 32 isolates. All of them tested sensitive to colistin, with MIC values between 0.25 and 1 µg/mL. Resistance to colistin, a last-resort antimicrobial for severe infections caused by MDR Gram-negative bacteria, represents a key concern for both public health and food safety. The absence of colistin-resistant isolates observed is in line with other studies, reporting resistance rates < 4% [43,44]; nonetheless, further studies should include these analyses on a greater number of isolates to provide a more comprehensive evaluation of resistance to colistin in poultry-associated *Salmonella*. Overall, the current findings highlight the widespread dissemination of high-priority resistance determinants and underline the importance of continued surveillance under a One Health perspective.

## 4. Materials and Methods

Between 2021 and 2025, a total number of 350 samples were analyzed, including 234 meat pieces/carcasses, 87 chicken skins, 21 fresh raw meat preparations, 7 frozen meat preparations and 1 cooked meat preparation related to food poisoning. Meat preparations included sausages, burger and breaded chicken/breaded rolls. The distribution of the different sample categories across the five study years is reported below:

- 2021: 41 meat pieces/carcasses,16 chicken skins, 2 fresh raw meat preparations;
- 2022: 51 meat pieces/carcasses, 17 chicken skins, 3 fresh raw meat preparations;
- 2023: 50 meat pieces/carcasses, 18 chicken skins, 5 fresh raw meat preparations;
- 2024: 47 meat pieces/carcasses, 18 chicken skins, 5 fresh raw meat preparations, 1 cooked meat preparation related to food poisoning;
- 2025: 45 meat pieces/carcasses, 18 chicken skins, 6 fresh raw meat preparations, 7 frozen meat preparations.

The sampling was carried out as part of the Annual Regional Control Plan (ARCP) of Sicily for microbiological risk and covered the whole geographical area of this region. The sampling events were independent and carried out on average 1–2 times a week, taking Sicilian food products at randomly selected commercial sites across the entire regional territory as well as samples collected at the three officially recognized slaughterhouses in Sicily, which process animals from all Sicilian poultry farms. Microbiological analyses were performed according to the criteria established by Regulation (EC) No 2073/2005, which defines mandatory limits for specific pathogens and hygiene indicators in food products. According to Reg. 2073/2005/EC, five sample units were tested for each sample, except for eight meat pieces/carcasses (only one unit per sample) and one skin sample (two units). In total, 1715 sample units were analyzed for *Salmonella*. A sample was considered positive when *Salmonella* spp. was detected in at least one sample unit.

**Figure 2:**
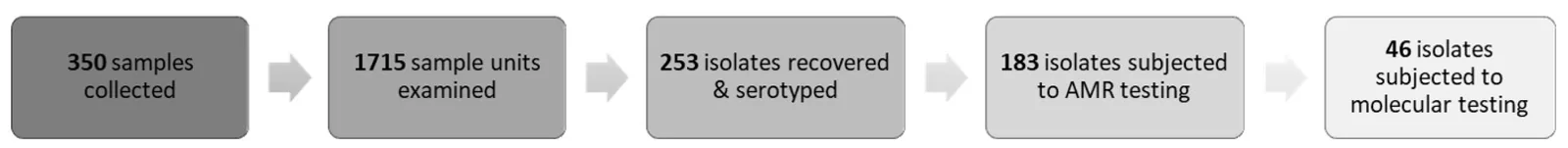
Flowchart depicting the numbers of samples collected, sample units examined, positive isolates recovered, isolates serotyped, and isolates subjected to antimicrobial susceptibility and molecular testing.

The flowchart given below (Figure 2) reports the numbers of samples collected, sample units examined, positive isolates recovered, isolates serotyped, and isolates subjected to antimicrobial susceptibility and molecular testing.

### 4.1. Bacteriological Analyses

The detection and isolation of *Salmonella* spp. was performed according to ISO 6579-1:2017/Amd 1:2020 [45] as follows: 25 g of sample was diluted 1:10 (*w*/*v*) in buffered peptone water (Oxoid, Milan, Italy), homogenized using a stomacher machine and incubated at 37 °C for 24 h (pre-enrichment). A 1:100 (*v*/*v*) dilution of the pre-enriched inoculum in 10 mL of Rappaport–Vassiliadis soya peptone broth (Oxoid, Milan, Italy) was incubated at 41.5 °C for 24 h. A 1:10 (*v*/*v*) dilution of the pre-enriched inoculum in 10 mL of Muller–Kauffmann tetrathionate novobiocin broth (Oxoid, Milan, Italy) was incubated at 37 °C for 24 h. Finally, a loopful of each inoculum was streaked onto two selective media plates, brilliant green agar modified and xylose lysine deoxycholate agar (Oxoid, Milan, Italy), and incubated at 37 °C for 24 h. Presumptive colonies were streaked onto nutrient agar (Oxoid, Milan, Italy) and incubated at 37 °C for 24 h. The oxidase-negative isolates were confirmed using miniaturized biochemical tests (API 20E, BioMerieux, Marcy l’Etoile, France). One *Salmonella* spp. isolate per sample unit was serotyped by direct slide agglutination, using specific antisera (Statens Serum Institut, Copenaghen, Denmark), according to the Kaufmann–White–Le Minor scheme and stored at −20 °C using Microbank^TM^ vials (Pro-Lab Diagnostics, Richmond Hill, ON, Canada) for subsequent analyses. Considering those samples included multiple positive units, one colony was recovered from each positive unit, resulting in more than one isolate obtained from one sample, e.g., 253 positive units out of 1715 units examined in total.

The antimicrobial susceptibility of the isolates was assessed by the Kirby–Bauer method (disc diffusion technique). The following 16 antimicrobials (Oxoid, Milan, Italy) were used: penicillins–ampicillin (AMP; 10 μg), amoxicillin/clavulanic acid (AMC; 20 μg/10 μg); third-generation cephalosporins–ceftazidime (CAZ; 30 μg), ceftriaxone (CRO, 30 µg), cefotaxime (CTX; 30 μg); carbapenems–imipenem (IMP; 10 μg); quinolones–nalidixic acid (NA; 30 µg); fluoroquinolones–ciprofloxacin (CIP; 5 μg), levofloxacin (LEV, 5 μg); aminoglycosides–kanamycin (K; 30 μg), streptomycin (S; 10 μg), gentamicin (CN; 10 μg), tobramycin (TOB, 10 μg); tetracyclines–tetracycline (TE; 30 μg); phenicols–chloramphenicol (C; 30 μg); pholate pathway antagonists–trimethoprim/sulfamethoxazole (STX; 25 μg). The diameters of the zones of inhibition surrounding each disk were measured using a digital caliper and interpreted according to the guidelines of the Clinical Laboratory Standards Institute [46,47]. The diameters of the inhibition zone (mm) corresponding to resistant/intermediate resistant isolates are reported as follows: AMP (≤13, 14–16); AMC (≤13, 14–17); CAZ (≤17, 18–20); CTX (≤22, 23–25); IMP (≤19, 20–22); NA30 (≤13, 14–18); CIP (≤20, 21–30); LEV (≤16; 17–20); K (≤13, 14–17); S (≤11, 12–14); CN (≤12, 13–14); TOB (≤12, 13–14); TE (≤11, 12–14); C (≤12, 13–17); SXT (≤10, 11–15).

Moreover, a subsample of 32 strains were tested for resistance to colistin using the broth microdilution method for the determination of minimum inhibitory concentration (MIC). In particular, the afore mentioned analyses included 29 *S.* Infantis (isolates 1, 5, 6, 8A,10A, 10B, 11A, 12, 17, 18, 20A, 26D, 31B, 32C, 36B, 38D, 39, 47, 50A, 55, 58, 70, 71, 72, 73, 78A, 82B, 84, 92) 1 *S.* Kentucky (isolate 9D) subjected also molecular testing and 2 *S.* Strathcona collected, respectively, in 2024 and in 2025. A suspension of bacteria (0,5 MacFarland in sterile saline solution) was transferred in pre-cast wells containing serial dilutions of colistin. The following final concentrations of antimicrobial were assessed (µg/mL): 0.25, 0.5, 1, 2, 4, 8, 16. Following incubation at 37 °C for 24 h, the MIC values were obtained and interpreted according to European Committee on Antimicrobial Susceptibility Testing (EUCAST) [48]. *E. coli* ATCC 25922 and *E. coli* NCTC 13846 were used as quality controls.

### 4.2. Molecular Analyses

Molecular analyses were performed on 46 *Salmonella* isolates selected for their antimicrobial susceptibility profiles. Among isolates belonging to the same serovar and recovered from the same sample, a single representative isolate was selected, prioritizing those R/I to a greater number of antimicrobials. Referring to the 12 isolates resistant/intermediately resistant to at least one fluoroquinolone, we could not recover viable bacteria from 4/12 Microbank^TM^ vials (Pro-Lab Diagnostics, Richmond Hill, ON, Canada) stored at −20 °C, hence only 8/12 isolates were subjected to molecular analyses. Among the 46 isolates selected, 4/46 were R/I solely to fluoroquinolones, 4/46 were R/I to fluoroquinolones and to third-generation cephalosporins, 38/46 were R/I solely to third-generation cephalosporins.

Genomic DNA was extracted according to the following protocol: one bead from each Microbank^TM^ vial (Pro-Lab Diagnostics, Richmond Hill, Canada) was streaked onto nutrient agar (Oxoid, Milan, Italy) and incubated at 37 °C for 24 h. Colonies were resuspended in 1 mL sterile nuclease-free water, centrifuged at 12,000 rpm for 1 min and resuspended in 200 µL of 6% *w*/*v* Chelex resin (InstaGene Matrix, Bio-rad, Hercules, CA, USA). The resuspension was first incubated at 56 °C for 20 min and subsequently at 100 °C ± 1 °C for 8 min. The supernatant was recovered following centrifugation at 12,000 rpm for 3 min. DNA quality and quantity were assessed using a NanoDrop microvolume spectrophotometer (LifeReal Nano-100, Life Real Biotechnology Co., Ltd., Hangzhou, China). The suitability of extracted DNA for downstream molecular analyses was preliminarily verified by amplification of the 16S rRNA gene using primers F1 and R12, as described before [49].

A PCR-based approach was used to screen for β-lactamase genes (*bla*_CTX-M-1_, _2_ and _4_, *bla*_TEM_, *bla*_OXA_ and *bla*_SHV_), plasmid-mediated AmpC (*bla*_CMY-2_ and *bla*_DHA_), plasmid-mediated quinolone resistance (PMQR) determinants (*qnrA*, *qnrB*, *qnrC*, *qnrD*, *qnrS*, *oqxA*, *oqxB*, *qepA)*, *aac(6′)-Ib*, and the class 1 integron integrase gene (*intI1*). The quinolone resistance-determining region (QRDR) of the *gyrA* gene was amplified by PCR, as described by Eaves et al. [50]. Amplicons were purified and sequenced in both forward and reverse directions by Sanger sequencing (BMR Genomics, Padova, Italy). Forward and reverse reads were assembled to generate a consensus sequence for each isolate. Consensus sequences were aligned to the *gyrA* reference sequence of *Salmonella enterica* serovar Typhimurium LT2 (GenBank accession number AE006468) to identify nucleotide and amino acid substitutions within the QRDR (positions 217–457; codons 73–152; Table S2). The primer sequences and expected amplicon size are listed in Table 4.

All PCR assays were performed using a final reaction volume of 25 µL, containing 2.5 µL of 10× DreamTaq Buffer (Thermo Fisher Scientific, Waltham, MA, USA), 0.5 µL of 10 µM forward and reverse primers, 0.5 µL of 10 µM dNTPs, 0.125 µL of 5 U/µL DreamTaq DNA Polymerase (Thermo Fisher Scientific, Waltham, MA, USA), 2 µL of DNA template, and nuclease-free water. The following thermal cycling conditions were applied [49]: initial denaturation step at 95 °C for 2 min, followed by 35 cycles of denaturation at 95 °C for 30 s, annealing at primer-specific temperatures (Table 4) for 30 s, elongation at 72 °C for 30–90 s (depending on the expected amplicon size), final elongation step at 72 °C for 5 min. The amplicons were analyzed by agarose gel electrophoresis (1–2% *w*/*v*) to verify amplification and amplicon size. Previously characterized isolates from our published studies served as positive controls for the CTX-M-1, CTX-M-2, CTX-M-4, DHA, TEM, OXA, SHV, and *qnrS* assays. Selected *intl1* amplicons were confirmed by Sanger sequencing, whereas sequencing of the expected-size *qnrD* products demonstrated nonspecific amplification.

### 4.3. Statistical Analyses

Data analysis was conducted using MS Excel 2019 version 1808. The prevalence of *Salmonella* spp. was calculated with 95% confidence interval (CI). The comparisons between sample categories and years were carried out by chi-squared test (meat pieces/carcasses vs. skins), chi-squared test with Yates continuity correction (comparisons between years) or Fisher exact test (skin vs. raw meat, meat pieces/carcasses vs. raw meat, frozen meat vs. skin). A probability value (*p*-value) of 0.05 was regarded as statistically significant.

## 5. Conclusions

This study provides a comprehensive overview of the prevalence of *Salmonella* spp. in poultry samples collected in Sicily between 2021 and 2025, together with the distribution of circulating serovars and AMR dissemination within these matrices. Our findings confirm poultry based-food as a significant source for the transmission of *Salmonella,* particularly the serovar *S*. Infantis. The high levels of antimicrobial resistance observed underscore the urgent need for continuous surveillance of *Salmonella* in poultry, especially considering critically important antimicrobials such as third-generation cephalosporins. Moreover, the molecular characterization of resistance determinants suggests potential worrying scenarios for the future safeguarding of human, animal, and environmental health, and highlights the need for targeted strategies to counteract this foodborne zoonosis.

## Figures and Tables

**Table 1:** Resistance to antimicrobial agents in *Salmonella* spp. strains.

Antimicrobial Classes	Antimicrobial	N. of Resistant Strains (%)	N. of Intermediate Resistant Strains (%)
Penicillins	Ampicillin	95 (51.9%)	12 (6.6%)
	Amoxicillin/clavulanic acid	58 (31.7%)	29 (15.8%)
3rd generation cephalosporins	Ceftazidime	13 (7.1%)	19 (10.4%)
	Ceftriaxone	50 (27.3%)	8 (4.4%)
	Cefotaxime	54 (29.5%)	8 (4.4%)
Carbapenems	Imipenem	0 (0%)	1 (0.5%)
Quinolones	Nalidixic acid	150 (82.0%)	3 (1.6%)
Fluoroquinolones	Ciprofloxacin	3 (1.6%)	8 (4.4%)
	Levofloxacin	1 (0.5%)	1 (0.5%)
Aminoglycosides	Kanamycin	41 (22.4%)	34 (18.6%)
	Streptomycin	26 (14.2%)	53 (29.0%)
	Gentamicin	3 (1.6%)	43 (23.5%)
	Tobramycin	12 (6.6%)	41 (22.4%)
Tetracyclines	Tetracycline	144 (78.7%)	7 (3.8%)
Phenicols	Chloramphenicol	9 (4.9%)	13 (7.1%)
Pholate pathway antagonists	Trimethoprim/sulfamethoxazole	126 (68.9%)	9 (4.9%)

**Table 2:** Resistance to single antimicrobial agents in the five most detected serovars.

AMR Classes	AMR	Infantis (n. = 153)	Kentucky (n. = 10)	Agona (n. = 5)	Enteritidis (n. = 4)	Strathcona (n. = 4)
Penicillins	AMP	R: 87 (56.9%), I: 11 (6.2%)	R: 1 (10.0%), I: 0 (0%)	R: 3 (60.0), I: 0 (0%)	R: 2 (50.0%), I: 1(25.0%)	R: 0 (0%), I: 0 (0%)
	AMC	R: 56 (36.6%), I: 26 (17.0%)	R: 0 (0%), I: 0 (0%)	R: 2 (40.0%), I: 1 (20.0%)	R: 0 (0%), I: 2 (50.0%)	R: 0 (0%), I: 0 (0%)
3rd generation cephalosporins	CAZ	R: 11 (7.2%), I: 19 (12.4%)	R: 1 (10.0%), I: 0 (0%)	R: 0 (0%), I: 0 (0%)	R: 0 (0%), I: 0 (0%)	R: 1 (25.0%), I: 0 (0%)
	CRO	R: 49 (32.0%), I: 8 (5.2%)	R: 1 (10.0%), I: 0 (0%)	R: 0 (0%), I: 0 (0%)	R: 0 (0%), I: 0 (0%)	R: 0 (0%), I: 0 (0%)
	CTX	R: 53 (34.6%), I: 7 (4.6%)	R: 1 (10.0%), I: 0 (0%)	R: 0 (0%), I: 0 (0%)	R: 0 (0%), I: 0 (0%)	R: 0 (0%), I: 0 (0%)
Carbapenems	IMP	R: 0 (0%), I: 1 (0.7%)	R: 0 (0%), I: 0 (0%)	R: 0 (0%), I: 0 (0%)	R: 0 (0%), I: 0 (0%)	R: 0 (0%), I: 0 (0%)
Quinolones	NA	R: 144 (94.1%), I: 0 (0%)	R: 1 (10.0%), I: 0 (0%)	R: 0 (0%), I: 0 (0%)	R: 3 (75.0%), I: 0 (0%)	R: 0 (0%), I: 3 (75.0%)
Fluoroquinolones	CIP	R: 3 (2.0%), I: 8 (5.2%)	R: 0 (0%), I: 0 (0%)	R: 0 (0%), I: 0 (0%)	R: 0 (0%), I: 0 (0%)	R: 0 (0%), I: 0 (0%)
	LEV	R: 1 (0.7%), I: 1 (0.7%)	R: 0 (0%), I: 0 (0%)	R: 0 (0%), I: 0 (0%)	R: 0 (0%), I: 0 (0%)	R: 0 (0%), I: 0 (0%)
Aminoglycosides	K	R: 41 (26.8%), I: 26 (17.0%)	R: 0 (0%), I: 4 (40.0%)	R: 0 (0%), I: 3 (60.0%)	R: 0 (0%), I: 0 (0%)	R: 0 (0%), I: 0 (0%)
	S	R: 24 (15.7%), I: 44 (28.8%)	R: 1 (10.0%), I: 4 (40.0%)	R: 0 (0%), I: 3 (60.0%)	R: 0 (0%), I: 0 (0%)	R: 1 (25.0%), I: 0 (0%)
	CN	R: 3 (2.0%), I: 32 (20.9%)	R: 0 (0%), I: 6 (60.0%)	R: 0 (0%), I: 3 (60.0%)	R: 0 (0%), I: 1 (25.0%)	R: 0 (0%), I: 0 (0%)
	TOB	R: 9 (5.9%), I: 32 (20.9%)	R: 0 (0%), I: 6 (60.0%)	R: 2 (40.0%), I: 3 (60.0%)	R: 0 (0%), I: 0 (0%)	R: 1 (25.0%), I: 0 (0%)
Tetracyclines	TE	R: 135 (88.2%), I: 2 (1.3%)	R: 3 (30.0%), I: 1 (10.0%)	R: 2 (40.0%), I: 0 (0%)	R: 1 (25.0%), I: 0 (0%)	R: 0 (0%), I: 3 (75.0%)
Phenicols	C	R: 7 (4.6%), I: 13 (8.5%)	R: 0 (0%), I: 0 (0%)	R: 2 (40.0%), I: 0 (0%)	R: 0 (0%), I: 0 (0%)	R: 0 (0%), I: 0 (0%)
Pholate pathway antagonists	SXT	R: 120 (78.4%), I: 6 (3.9%)	R: 1 (10.1%), I: 0 (0%)	R: 3 (60.0%), I: 0 (0%)	R: 0 (0%), I: 0 (0%)	R: 0 (0%), I: 3 (75.0%)

AMR: antimicrobial, R: resistant, I: intermediate resistant, AMP: ampicillin, AMC: amoxicillin/clavulanic acid, CAZ: ceftazidime, CRO: ceftriaxone, CTX: cefotaxime, IMP: imipenem, NA: nalidixic acid, CIP: ciprofloxacin, LEV: levofloxacin, K: kanamycin, S: streptomycin, CN: gentamicin, TOB: tobramycin, TE: tetracycline, C: chloramphenicol, SXT: trimethoprim/sulfamethoxazole.

**Table 3:** Antimicrobial susceptibility phenotypes and associated genetic determinants in *Salmonella* isolates.

Isolate ID	AMR Profile	Resistance Determinants		
		β-Lactam	Quinolone	Integron-Associated
1	AMP R; AMC I; CAZ S; CRO S; CTX R; IMP S; NA R; CIP S; LEV R, K R; S R; CN S; TOB S; TE S; C R; SXT R	*bla*_CTX-M-1_, *bla*_CTX-M-2_,	*gyrA D87G*	*intI1*
4	AMP R; AMC S; CAZ S; CRO S; CTX S; IMP S; NA R; CIP I; LEV S; K R; S R; CN S; TOB I; TE R; C I; SXT R	NT	*gyrA D87G*	*intI1*
5	AMP R; AMC S; CAZ I; CRO R; CTX R; IMP S; NA R; CIP S; LEV S; K R; S S, CN I; TOB R; TE S; C S; SXT R	*bla*_CTX-M-1_, *bla*_CTX-M-2_	NT	*intI1*
6	AMP R; AMC R; CAZ S; CRO R; CTX R; IMP S; NA R; CIP S; LEV S; K S; S S; CN S; TOB S; TE R; C I; SXT R	*bla*_CTX-M-1_, *bla*_CTX-M-2_	NT	*intI1*
7	AMP R; AMC S; CAZ S; CRO S; CTX S; IMP S; NA R; CIP I; LEV S; K S; S R; CN I; TOB I; TE R; C S; SXT S	NT	*gyrA D87G*	*intI1*
8A *	AMP R; AMC R; CAZ S; CRO R; CTX R; IMP S; NA R; CIP S; LEV S; K R; S R; CN S; TOB R; TE R; C R; SXT R	*bla*_CTX-M-1_, *bla*_CTX-M-2_	NT	*intI1*
9D *	AMP R; AMC S; CAZ R; CRO R; CTX R; IMP S; NA R; CIP S; LEV S; K I; S R; CN I; TOB I; TE R; C S; SXT R	NT	NT	*intI1*
9E *	AMP R; AMC I; CAZ R; CRO R; CTX R; IMP S; NA R; CIP S; LEV S; K I; S S; CN I; TOB I; TE R; C S; SXT R	ND	NT	*intI1*
10A *	AMP S; AMC R; CAZ S; CRO R; CTX R; IMP S; NA S; CIP R; LEV S; K R; S I; CN R; TOB I; TE S; C R; SXT R	*bla*_CTX-M-1_, *bla*_CTX-M-2_	*gyrA S83Y*	*intI1*
10B *	AMP R; AMC R; CAZ I; CRO R; CTX R; IMP S; NA R; CIP S; LEV S; K I; S I; CN S; TOB R; TE R; C S; SXT R	*bla*_CTX-M-1_, *bla*_CTX-M-2_	NT	*intI1*
11A *	AMP R; AMC S; CAZ I; CRO R; CTX R; IMP S; NA R; CIP S; LEV S; K S; S S; CN R; TOB I; TE R; C S; SXT R	*bla*_CTX-M-1_, *bla*_CTX-M-2_	NT	*intI1*
11B *	AMP R; AMC S; CAZ S; CRO S; CTX S; IMP S; NA R; CIP I; LEV S; K R; S S; CN I; TOB R; TE R; C I; SXT R	NT	*gyrA D87G*	*intI1*
12	AMP R; AMC I; CAZ R; CRO S; CTX R; IMP S; NA R; CIP S; LEV S; K I; S S; CN I; TOB I; TE R; C S; SXT R	*bla*_CTX-M-1_, *bla*_CTX-M-2_	NT	*intI1*
17	AMP R; AMC R; CAZ S; CRO R; CTX S; IMP S; NA R; CIP S; LEV S; K R; S S; CN I; TOB I; TE R; C S; SXT R	*bla*_CTX-M-1_, *bla*_CTX-M-2_	NT	*intI1*
18	AMP R; AMC R; CAZ S; CRO S; CTX R; IMP S; NA R; CIP S; LEV S; K R; S S; CN S; TOB R; TE R; C S; SXT R	*bla*_CTX-M-1_, *bla*_CTX-M-2_	NT	*intI1*
20A *	AMP R; AMC R; CAZ S; CRO R; CTX R; IMP S; NA R; CIP S; LEV S; K I; S I; CN I; TOB I; TE R; C S; SXT R	*bla*_CTX-M-1_, *bla*_CTX-M-2_	NT	*intI1*
26D *	AMP R; AMC R; CAZ S; CRO R; CTX R; IMP S; NA R; CIP S; LEV S; K R; S I; CN I; TOB I; TE R; C S; SXT R	*bla*_CTX-M-1_, *bla*_CTX-M-2_	NT	*intI1*
26E *	AMP R; AMC R; CAZ S; CRO R; CTX R; IMP S; NA R; CIP I; LEV S; K R; S I; CN S; TOB S; TE R; C S; SXT R	*bla*_CTX-M-1_, *bla*_CTX-M-2_	*gyrA D87G*	*intI1*
31B *	AMP R; AMC R; CAZ S; CRO R; CTX R; IMP S; NA R; CIP S; LEV S; K R; S S; CN S; TOB S; TE R; C S; SXT R	*bla*_CTX-M-1_, *bla*_CTX-M-2_	NT	*intI1*
32C *	AMP R; AM R; CAZ I; CRO R; CTX R; IMP S; NA R; CIP S; LEV S; K I; S R; CN S; TOB S; TE R; C S; SXT R	*bla*_CTX-M-1_, *bla*_CTX-M-2_	NT	*intI1*
36B *	AMP R; AM R; CAZ I; CRO R; CTX R; IMP S; NA R; CIP S; LEV S; K R; S I; CN S; TOB S; TE R; C R; SXT R	*bla*_CTX-M-1_, *bla*_CTX-M-2_	NT	*intI1*
37A *	AMP R; AM R; CAZ S; CRO R; CTX R; IMP S; NA R; CIP S; LEV S; K S; S I; CN I, TOB S, TE R; C S; SXT R	*bla*_TEM_	NT	*intI1*
38D *	AMP R; AM R; CAZ I; CRO R; CTX R; IMP S; NA R; CIP S; LEV S; K R; S I; CN S; TOB S; TE R; C S, SXT R	*bla*_CTX-M-1_	NT	*intI1*
39	AMP R; AMC I; CAZ S; CRO R; CTX R; IMP S; NA R; CIP S; LEV S; K R; S S; CN S; TOB S; TE R; C S; SXT R	*bla*_CTX-M-1_, *bla*_CTX-M-2_	NT	*intI1*
47	AMP R; AMC R; CAZ S; CRO R; CTX R; IMP S; NA R; CIP S; LEV S; K S; S S; CN S; TOB S; TE R; C S; SXT R	*bla*_CTX-M-1_, *bla*_CTX-M-2_	NT	*intI1*
50A *	AMP R; AMC R; CAZ S; CRO R; CTX R; IMP S; NA R; CIP S; LEV S; K S; S S; CN S; TOB S; TE R; C S; SXT S	*bla*_CTX-M-1_, *bla*_CTX-M-2_	NT	*intI1*
55	AMP R; AMC R; CAZ S; CRO R; CTX R; IMP S; NA R; CIP S; LEV S; K S; S S; CN S; TOB S; TE R; C I; SXT R	*bla*_CTX-M-1_, *bla*_CTX-M-2_	NT	*intI1*
57B *	AMP S; AMC S; CAZ R; CRO S; CTX S; IMP S; NA R; CIP S; LEV S; K R; S S; CN S; TOB S; TE R; C S; SXT R	ND	NT	*intI1*
58	AMP R; AMC R; CAZ S; CRO R; CTX R; IMP S; NA R; CIP S; LEV S; K R; S S; CN S; TOB S; TE R; C S; SXT R	*bla*_CTX-M-1_, *bla*_CTX-M-2_	NT	*intI1*
59A *	AMP S; AMC S; CAZ R; CRO S; CTX S; IMP S; NA R; CIP S; LEV S; K S; S S; CN S; TOB S; TE R; C S; SXT R	ND	NT	*intI1*
60A *	AMP S; AMC S; CAZ R; CRO S; CTX S; IMP S; NA R; CIP S; LEV S; K R; S S; CN S; TOB S; TE R; C S; SXT R	ND	NT	*intI1*
62A *	AMP S; AMC S; CAZ R; CRO S; CTX S; IMP S; NA R; CIP I; LEV S; K S; S S; CN I; TOB S; TE R; C S; SXT R	NT	*gyrA D87G*	*intI1*
63	AMP S; AMC S; CAZ I; CRO S; CTX S; IMP S; NA R; CIP I; LEV S; K S; S I; CN S; TOB S; TE R; C S; SXT R	ND	*gyrA D87G*	*intI1*
70	AMP R; AMC R; CAZ S; CRO R; CTX R; IMP S; NA R; CIP S; LEV S; K R; S S; CN S; TOB S; TE R; C S; SXT I	*bla*_CTX-M-1_, *bla*_CTX-M-2_	NT	*intI1*
71	AMP R; AMC R; CAZ S; CRO R; CTX R; IMP S; NA R; CIP S; LEV S; K S; S S; CN S; TOB S; TE R; C S; SXT R	*bla*_CTX-M-1_, *bla*_CTX-M-2_	NT	*intI1*
72	AMP R; AMC R; CAZ S; CRO R; CTX R; IMP S; NA R; CIP S; LEV S; K S; S S; CN S; TOB S; TE R; C S; SXT R	*bla*_CTX-M-1_, *bla*_CTX-M-2_	NT	*intI1*
73	AMP R; AMC R; CAZ S; CRO R; CTX R; IMP S; NA R; CIP S; LEV S; K S; S S; CN S; TOB S; TE R; C S; SXT R	*bla*_CTX-M-1_, *bla*_CTX-M-2_	NT	*intI1*
78A *	AMP R; AMC R; CAZ I; CRO I; CTX R; IMP S; NA R; CIP S; LEV S; K I; S R; CN I; TOB S; TE R; C I; SXT R	*bla*_CTX-M-1_,	NT	*intI1*
82B *	AMP R; AMC R; CAZ S; CRO R; CTX R; IMP S; NA R; CIP S; LEV S; K S; S S; CN S; TOB S; TE R; C S; SXT R	*bla*_CTX-M-1_, *bla*_CTX-M-2_	NT	*intI1*
84	AMP R; AMC R; CAZ I; CRO R; CTX R IMP S; NA R; CIP S; LEV S; K R; S S; CN S; TOB S; TE R; C S; SXT R	*bla*_CTX-M-1_, *bla*_CTX-M-2_	NT	*intI1*
88A *	AMP R; AMC R; CAZ S; CRO R; CTX R; IMP S; NA R; CIP S; LEV S; K S; S S; CN S; TOB S; TE R; C S; SXT R	*bla*_CTX-M-1_	NT	*intI1*
90	AMP R; AMC R; CAZ I; CRO R; CTX R IMP S; NA R; CIP S; LEV S; K S; S I; CN S; TOB S; TE R; C S; SXT R	*bla*_CTX-M-2_	NT	*intI1*
92	AMP R; AMC R; CAZ R; CRO R; CTX R; IMP S; NA R; CIP S; LEV S; K S; S R; CN S; TOB S; TE S; C S; SXT R	*bla*_CTX-M-1_, *bla*_CTX-M-2_	NT	*intI1*
97	AMP S; AMC S; CAZ R; CRO S; CTX S; IMP S; NA R; CIP S; LEV S; K S; S I; CN S; TOB S; TE R; C S; SXT S	ND	NT	*intI1*
98	AMP S; AMC S; CAZ R; CRO S; CTX S; IMP S; NA R; CIP S; LEV S; K S; S I; CN S; TOB S; TE R; C S; SXT R	ND	NT	*intI1*
99B *	AMP S; AMC S; CAZ R; CRO S; CTX S; IMP S; NA R; CIP S; LEV S; K R; S S; CN S; TOB S; TE R; C S; SXT R	ND	NT	*intI1*

* When more-than-one sample unit per sample tested positive, the isolates were given an ID that included a number (which refers to the source sample’s ID reported at Table S1, column 2) and a letter (which refers to the sample unit). AMR: antimicrobial, R: resistant, I: intermediate resistant, AMP: ampicillin, AMC: amoxicillin/clavulanic acid, CAZ: ceftazidime, CRO: ceftriaxone, CTX: cefotaxime, IMP: imipenem, NA: nalidixic acid, CIP: ciprofloxacin, LEV: levofloxacin, K: kanamycin, S: streptomycin, CN: gentamicin, TOB: tobramycin, TE: tetracycline, C: chloramphenicol, SXT: trimethoprim/sulfamethoxazole. R: resistant, I: intermediate resistant, S: susceptible. Resistance at the class level was calculated considering the number of isolates resistant to at least one of the antimicrobials tested from that class. NT: not tested for the corresponding determinant category; ND: none of the screened determinants in that category detected.

**Table 4:** Primer sequences used for PCR assays and expected amplicon size.

Target Gene	Primer Sequence (5’–3’)	T_a_ (°C)	Amplicon Size (bp)	Reference
*16S* rRNA gene	F: GAGTTTGATCCTGGCTCAG R: ACGGCTACCTTGTTACGACT	56	1402	[49]
CTX-M-1	F: TCCAGAATAAGGAATCCCATGG R: TGCTTTACCCAGCGTCAGAT	61	621	
CTX-M-2	F: ACCGCCGATAATTCGCAGAT R: GATATCGTTGGTGGTGCCATAA	61	588	
CTX-M-4	F: GACAAAGAGAGTGCAACGGATG R: TCAGTGCGATCCAGACGAAA	61	501	
TEM	F: AGTGCTGCCATAACCATGAGTG R: CTGACTCCCCGTCGTGTAGATA	61	431	
OXA	F: ATTATCTACAGCAGCGCCAGTG R: TGCATCCACGTCTTTGGTG	61	296	
SHV	F: GATGAACGCTTTCCCATGATG R: CGCTGTTATCGCTCATGGTAA	61	214	
CMY-2	F: AGCGATCCGGTCACGAAATA R: CCCGTTTTATGCACCCATGA	61	695	
DHA	F: GTGGTGGACAGCACCATTAAA R: CCTGCGGTATAGGTAGCCAGAT	61	314	
*qnrA*	F: ATTTCTCACGCCAGGATTTG R: TGCCAGGCACAGATCTTGAC	60	516	
*qnrB*	F: CGACCTKAGCGGCACTGAAT R: GAGCAACGAYGCCTGGTAGYTG	56	515	
*qnrC*	F: GGGTTGTACATTTATTGAATC R: TCCACTTTACGAGGTTCT	50	446	
*qnrD*	F: CGAGATCAATTTACGGGGAATA R: AACAAGCTGAAGCGCCTG	60	581	
*qnrS*	F: GACGTGCTAACTTGCGTGAT R: TGGCATTGTTGGAAACTTG	58	118	
*oqxA*	F: CTCGGCGCGATGATGCT R: CCACTCTTCACGGGAGACGA	60	392	[51]
*oqxB*	F: TTCTCCCCCGGCGGGAAGTAC R: CTCGGCCATTTTGGCGCGTA	64	512	
*qepA*	F: GCAGGTCCAGCAGCGGGTAG R: CTTCCTGCCCGAGTATCGTG	60	199	[11]
*aac(6’)-lb*	F: TTGCGATGCTCTATGAGTGGCTA R: CTCGAATGCCTGGCGTGTTT	55	482	[52]
*intI1*	F: GGCTTCGTGATGCCTGCTT R: CATTCCTGGCCGTGGTTCT	59	148	[53]
*gyrA*	F: CGTTGGTGACGTAATCGGTA R: CCGTACCGTCATAGTTATCC	53	251	[50]

## Data Availability

The original contributions presented in this study are included in the article. Further inquiries can be directed to the corresponding authors.

## Supplementary Materials

The following supporting information can be downloaded at: https://www.mdpi.com/article/10.3390/ijms27219876/s1.
