# Supplementary material for: Salmonella Serovars Distribution in Poultry Meat and Genetic Characterization of Antimicrobial-Resistant Isolates: A Five-Year Monitoring in Sicily
Source: Int J Mol Sci. 2026 Nov 4;27(21):9876. doi: 10.3390/ijms27219876 (PMC13764154; doi:10.3390/ijms27219876)
Supplement: Supplementary file 1 [file ijms-27-09876-s001.zip › ijms-4568817-supplementary.pdf]

Table S1. Samples positive for *Salmonella* spp., number of sample units testing positive per sample, and detected serovars.

Year	ID	Source	Serovars detected (n. of sample units tested positive)
2021	1	meat pieces/carcasses	*S.* Infantis (2/5)
	2	meat pieces/carcasses	*S.* Agona (2/5)
	3	meat pieces/carcasses	*S.* Infantis (1/5)
	4	meat pieces/carcasses	*S.* Infantis (1/5)
	5	meat pieces/carcasses	*S.* Infantis (1/5)
	6	meat pieces/carcasses	*S.* Infantis (1/5)
	7	meat pieces/carcasses	*S.* Infantis (4/5)
	8	skin	*S.* Infantis (3/5)
	9	meat pieces/carcasses	*S.* Infantis (3/5), *S.* Kentucky (2/5)
	10	meat pieces/carcasses	*S.* Infantis (3/5)
	11	meat pieces/carcasses	*S.* Infantis (4/5)
	12	meat pieces/carcasses	*S.* Infantis (1/1)
	13	meat pieces/carcasses	*S.* Infantis (1/1)
	14	meat pieces/carcasses	*S.* Infantis (1/5)
2022	15	raw meat preparation	*S.* Infantis (5/5)
	16	meat pieces/carcasses	*S.* Infantis (2/5)
	17	meat pieces/carcasses	*S.* Infantis (1/5)
	18	skin	*S.* Infantis (1/5)
	19	meat pieces/carcasses	*S.* Infantis (1/5)
	20	meat pieces/carcasses	*S.* Infantis (4/5)
	21	meat pieces/carcasses	*S.* Agona (3/5)
	22	meat pieces/carcasses	*S.* Infantis (4/5)
	23	meat pieces/carcasses	*S.* Infantis (4/5)
	24	meat pieces/carcasses	*S.* Enteritidis (1/5), *S.* Infantis (1/5)
	25	meat pieces/carcasses	*S.* Infantis (2/5)
	26	skin	*S.* Infantis (5/5)
	27	skin	*S.* Kentucky (1/5)
	28	skin	*S.* Kentucky (3/5)
	29	meat pieces/carcasses	*S.* Infantis (1/5)
	30	meat pieces/carcasses	*S.* Infantis (5/5)
	31	skin	*S.* Infantis (5/5)
	32	skin	*S.* Infantis (3/5), *S.* Toulon (2/5)
	33	meat pieces/carcasses	*S.* Infantis (1/5)
	34	skin	*S.* Infantis (2/5)
	35	meat pieces/carcasses	*S.* Infantis (1/5)
	36	meat pieces/carcasses	*S.* Infantis (2/5)
	37	raw meat preparation	*S.* Infantis (2/5)
	38	meat pieces/carcasses	*S.* Infantis (4/5)
	39	skin	*S.* Infantis (1/5)
	40	skin	*S.* Kentucky (4/5)
2023	41	meat pieces/carcasses	*S.* Infantis (1/5)
	42	skin	*S.* Infantis (4/5)
	43	skin	*S.* Infantis (2/2)
	44	meat pieces/carcasses	*S.* Infantis (5/5)

	45	skin	*S.* Infantis (4/5)
	46	meat pieces/carcasses	*S.* Infantis (1/5)
	47	skin	*S.* Infantis (1/5)
	48	skin	*S.* Infantis (2/5)
	49	meat pieces/carcasses	*S.* Enteritidis (1/5)
	50	skin	*S.* Infantis (2/5), *S.* Kentucky (2/5)
	51	skin	*S.* Infantis (1/5)
	52	skin	*S.* Corvallis (1/5), *S.* Infantis (4/5)
	53	meat pieces/carcasses	*S.* Infantis (5/5)
	54	skin	*S.* Corvallis (2/5), *S.* Infantis (3/5)
	55	raw meat preparation	*S.* Infantis (1/5)
	56	skin	*S.* Infantis (4/5)
	57	meat pieces/carcasses	*S.* Infantis (4/5)
	58	skin	*S.* Infantis (2/5)
	59	skin	*S.* Infantis (5/5)
	60	meat pieces/carcasses	*S.* Infantis (4/5)
	61	skin	*S.* Infantis (2/5)
2024	62	skin	*S.* Infantis (2/5)
	63	meat pieces/carcasses	*S.* Infantis (1/5)
	64	meat pieces/carcasses	*S.* Infantis (2/5)
	65	skin	*S.* Infantis (1/5)
	66	meat pieces/carcasses	*S.* Infantis (3/5)
	67	meat pieces/carcasses	*S.* Infantis (1/5)
	68	meat pieces/carcasses	*S.* Infantis (5/5)
	69	meat pieces/carcasses	*S.* Infantis (1/5)
	70	skin	*S.* Infantis (1/5)
	71	meat pieces/carcasses	*S.* Infantis (2/5)
	72	meat pieces/carcasses	*S.* Infantis (2/5)
	73	meat pieces/carcasses	*S.* Infantis (1/5)
	74	skin	*S.* Infantis (2/5)
	75	skin	*S.* Infantis (5/5)
	76	meat pieces/carcasses	*S.* Infantis (1/5)
	77	skin	*S.* Infantis (1/5)
	78	meat pieces/carcasses	*S.* Infantis (5/5)
	79	meat preparation- food poisoning	*S.* Enteritidis (2/5)
	80	skin	*S.* Virchow (2/5)
	81	skin	*S.* Strathcona (1/5)
	82	skin	*S.* Infantis (5/5)
	83	skin	*S.* Infantis (1/5)
2025	84	skin	*S.* Infantis (1/5)
	85	meat pieces/carcasses	*S.* Infantis (1/5)
	86	meat pieces/carcasses	*S.* Infantis (1/5)
	87	meat pieces/carcasses	*S.* Infantis (1/5)
	88	skin	*S.* Infantis (3/5)
	89	meat pieces/carcasses	*S.* Infantis (5/5)
	90	meat pieces/carcasses	*S.* Infantis (1/5)
	91	skin	*S.* Infantis (1/5)
	92	meat pieces/carcasses	*S.* Infantis
	93	skin	*S.* Infantis
	94	frozen meat preparation	*S.* Infantis (1/5)
	95	frozen meat preparation	*S.* Infantis
	96	frozen meat preparation	*S.* Infantis (1/5)

97	frozen meat preparation	*S.* Infantis (1/5)
98	frozen meat preparation	*S.* Infantis (2/5)
99	frozen meat preparation	*S.* Infantis (3/5)
100	raw meat preparation	*S.* Infantis (5/5)
101	Skin	*S.* Strathcona (5/5)

Table S2. Consensus nucleotide sequences of the *gyrA* quinolone resistance–determining region (QRDR) and amino acid substitutions identified in eight *Salmonella* isolates.

ID	Consensus sequence (5′- 3′)	Identified mutation
Reference - *S. Typhimurium* LT2, accession AE006468.	CGTTGGTGACGTAATCGGTAAATACCATCCC<br>CACGGCGATTCCGCAGTGTATGACACCATCG<br>TTCGTATGGCGCAGCCATTCTCGCTGCGTTA<br>CATGCTGGTGGATGGTCAGGGTAACTTCGGT<br>TCTATTGACGGCGACTCCGCGGCGGCAATGC<br>GTTATACGGAGATCCGTCTGGCGAAAATCGC<br>CCACGAACTGATGGCCGATCTCGAAAAAGA<br>GACGGTGGATTTCGTGGATAACTATGACGGT<br>ACGG	-
1	GTAATCGGTAAATACCATCCCCACGGCGATT<br>CCGCAGTGTATG**G**CACCATCGTTCGTATGGC<br>GCAGCCATTCTCGCTGCGTTACATGCTGGTG<br>GATGGTCAGGGTAACTTCGGTTCTATTGACG<br>GCGACTCCGCGGCGGCAATGCGTTATACGG<br>AGATCCGTCTGGCGAAAATCGCCCACGAAC<br>TGATGGCCGATCTCGAAAAAGAGACGGTGG<br>ATTTCGTGGATAACTATGACGGTACGG	**D87G** - GAC→GGC
4	GTTGGTGACGTAATCGGTAAATACCATCCCC<br>ACGGCGATTCCGCAGTGTATG**G**CACCATCGT<br>TCGTATGGCGCAGCCATTCTCGCTGCGTTAC<br>ATGCTGGTGGATGGTCAGGGTAACTTCGGTT<br>CTATTGACGGCGACTCCGCGGCGGCAATGC<br>GTTATACGGAGATCCGTCTGGCGAAAATCGC<br>CCACGAACTGATGGCCGATCTCGAAAAAGA<br>GACGGTGGATTTCGTGGATAACTATGACGGT<br>ACGG	**D87G** - GAC→GGC
7	GTTGGTGACGTAATCGGTAAATACCATCCCC<br>ACGGCGATTCCGCAGTGTATG**G**CACCATCGT<br>TCGTATGGCGCAGCCATTCTCGCTGCGTTAC<br>ATGCTGGTGGATGGTCAGGGTAACTTCGGTT<br>CTATTGACGGCGACTCCGCGGCGGCAATGC<br>GTTATACGGAGATCCGTCTGGCGAAAATCGC<br>CCACGAACTGATGGCCGATCTCGAAAAAGA<br>GACGGTGGATTTCGTGGATAACTATGACGGT<br>ACGG	**D87G** - GAC→GGC

ID	Consensus sequence (5′- 3′)	Identified mutation
10A*	CGTTGGTGACGTAATCGGTAAATACCATCCC<br>CACGGCGATT**A**CGCAGTGTATGACACCATCG<br>TTCGTATGGCGCAGCCATTCTCGCTGCGTTA<br>CATGCTGGTGGATGGTCAGGGTAACTTCGGT<br>TCTATTGACGGCGACTCCGCGGCGGCAATGC<br>GTTATACGGAGATCCGTCTGGCGAAAATCGC<br>CCACGAACTGAKGSCSKMNSWMRAAAAAGA<br>GACGGTGGATTTCGTGGATAACTATGACGGT<br>ACGG	**S83Y** - TCC→TAC
11B*	GTTGGTGACGTAATCGGTAAATACCATCCCC<br>ACGGCGATTCCGCAGTGTATG**G**CACCATCGT<br>TCGTATGGCGCAGCCATTCTCGCTGCGTTAC<br>ATGCTGGTGGATGGTCAGGGTAACTTCGGTT<br>CTATTGACGGCGACTCCGCGGCGGCAATGC<br>GTTATACGGAGATCCGTCTGGCGAAAATCGC<br>CCACGAACTGATGGCCGATCTCGAAAAAGA<br>GACGGTGGATTTCGTGGATAACTATGACGGT<br>ACGG	**D87G** - GAC→GGC
26E*	CGTTGGTGACGTAATCGGTAAATACCATCCC<br>CACGGCGATTCCGCAGTGTATG**G**CACCATCG<br>TTCGTATGGCGCAGCCATTCTCGCTGCGTTA<br>CATGCTGGTGGATGGTCAGGGTAACTTCGGT<br>TCTATTGACGGCGACTCCGCGGCGGCAATGC<br>GTTATACGGAGATCCGTCTGGCGAAAATCGC<br>CCACGAACTGATGGCCGATCTCGAAAAAGA<br>GACGGTGGATTTCGTGGATAACTATGACGGT<br>ACGG	**D87G** - GAC→GGC
62A*	GTTGGTGACGTAATCGGTAAATACCATCCCC<br>ACGGCGATTCCGCAGTGTATG**G**CACCATCGT<br>TCGTATGGCGCAGCCATTCTCGCTGCGTTAC<br>ATGCTGGTGGATGGTCAGGGTAACTTCGGTT<br>CTATTGACGGCGACTCCGCGGCGGCAATGC<br>GTTATACGGAGATCCGTCTGGCGAAAATCGC<br>CCACGAACTGATGGCCGATCTCGAAAAAGA<br>GACGGTGGATTTCGTGGATAACTATGACGGT<br>ACGG	**D87G** - GAC→GGC
63	CGTTGGTGACGTAATCGGTAAATACCATCCC<br>CACGGCGATTCCGCAGTGTATG**G**CACCATCG<br>TTCGTATGGCGCAGCCATTCTCGCTGCGTTA<br>CATGCTGGTGGATGGTCAGGGTAACTTCGGT<br>TCTATTGACGGCGACTCCGCGGCGGCAATGC<br>GTTATACGGAGATCCGTCTGGCGAAAATCGC<br>CCACGAACTGATGGCCGATCTCGAAAAAGA<br>GACGGTGGATTTCGTGGATAACTATGACGGT<br>ACGG	**D87G** - GAC→GGC

Sequences are shown in the 5′-3′ orientation. Substitutions are reported relative to *Salmonella enterica* serovar Typhimurium LT2 (GenBank accession AE006468). Bold nucleotides indicate the substitutions underlying S83Y (TCC→TAC) or D87G (GAC→GGC). The shared region analyzed spans *gyrA* nucleotide positions 217–457 (complete codons 73–152), with nucleotide numbering starting from the first base of the start codon.
